# 3D Analysis of D-RaCe and Self-Adjusting File in Removing Filling Materials from Curved Root Canals Instrumented and Filled with Different Techniques

**DOI:** 10.1155/2014/836513

**Published:** 2014-07-09

**Authors:** Neslihan Simsek, Fuat Ahmetoglu, Ali Keles, Elcin Tekin Bulut, Kursat Er

**Affiliations:** ^1^Department of Endodontics, Faculty of Dentistry, Inonu University, 44280 Malatya, Turkey; ^2^Department of Endodontics, Faculty of Dentistry, Akdeniz University, 07058 Antalya, Turkey

## Abstract

The aim of this study was to compare the efficacy of D-RaCe files and a self-adjusting file (SAF) system in removing filling material from curved root canals instrumented and filled with different techniques by using microcomputed tomography (micro-CT). The mesial roots of 20 extracted mandibular first molars were used. Root canals (mesiobuccal and mesiolingual) were instrumented with SAF or Revo-S. The canals were then filled with gutta-percha and AH Plus sealer using cold lateral compaction or thermoplasticized injectable techniques. The root fillings were first removed with D-RaCe (Step 1), followed by Step 2, in which a SAF system was used to remove the residual fillings in all groups. Micro-CT scans were used to measure the volume of residual filling after root canal filling, reinstrumentation with D-RaCe (Step 1), and reinstrumentation with SAF (Step 2). Data were analyzed using Wilcoxon and Kruskal-Wallis tests. There were no statistically significant differences between filling techniques in the canals instrumented with SAF (*P* = 0.292) and Revo-S (*P* = 0.306). The amount of remaining filling material was similar in all groups (*P* = 0.363); all of the instrumentation techniques left filling residue inside the canals. However, the additional use of SAF was more effective than using D-RaCe alone.

## 1. Introduction

A fundamental aim of endodontic treatment is to maintain the long-term health and function of teeth. When preparing root canals for the treatment, it is essential to prevent possible reinfection. Successful endodontic treatment, therefore, requires cleaning, shaping, and total three-dimensional filling of the root canal systems [1].

While initial root canal treatment has the potential for a successful outcome, with high survival rates of treated teeth [2], failure is also a possibility (12–19%) [2, 3]. Some causes of endodontic treatment failure are perforation, inadequate sealing of missed canals, unresolved cystic lesions, iatrogenic manipulation, radicular fracture, or reinfection of the root canal system [1, 3].

Nonsurgical retreatment, apical surgery, and intentional reimplantation are possible solutions to failed root canal treatment. Because nonsurgical retreatment is the least invasive approach, it is the favored treatment choice [4]. The main objective of this treatment is to remove all filling materials from the canal and regain access to the apical foramen. Gutta-percha, in combination with a variety of sealers, is the most commonly used material for canal filling; however, it cannot be removed completely from root canals when retreatment is required. Attempts to do so have been made with hand [5] and rotary [6] files, ultrasonic systems [7], heat carrying instruments [8], lasers [9], and solvents [10]. Some studies [2, 3] have reported endodontic retreatment success rates of 62%–82% when evaluated for radiolucency, filling quality, and perforation.

Various nickel-titanium (NiTi) rotary files have been developed to remove fillings from canal walls. To improve safety preparation and prepare the intended shapes, new file designs with cutting tips, radial lands, varying taper and rake angles, and varying pitch lengths have been developed. The commercial D-RaCe file system (FKG Dentaire, La Chaux-de-Fonds, Switzerland), which has recently been introduced, consists of two files: DR1, with an active tip (size 30) and .10 taper, and DR2, with a nonactive tip (size 25) and .04 taper. The files were designed with a triangular cross-section and alternating cutting edges; the length of the DR1 is 15 mm, and the length of the DR2 is 25 mm. These files were specifically designed for the removal of filling material from the coronal (with DR1), middle, and apical thirds (with DR2) of root canals. In addition, the DR1 working tip facilitates the initial penetration into the filling. da Silva et al. [4] reported that D-RaCe is less effective when compared to ProTaper, while Rödig et al. [11] found that D-RaCe is more effective and faster than ProTaper.

A newly developed instrumentation and irrigation device, the self-adjusting file (SAF) system, was introduced by ReDent-Nova (Ra'anana, Israel). It uses a hollow reciprocating instrument that allows simultaneous irrigation throughout the mechanical preparation process. When inserted into a root canal, the SAF adapts itself to the canal's shape longitudinally and in cross-section. It operates in a transline (in-and-out) vibrating motion, and coupled with the abrasive surface of the lattice threads, it promotes uniform removal of dentin [12]. Peters and Paque [13] also showed that the SAF system is capable of touching all the canal walls. Effects of the SAF on residual root filling were evaluated in several studies [14–17] and were found to be significantly effective.

The aim of this study was to compare, using microcomputed tomography (micro-CT), the efficacy of D-RaCe with the combined instrumentation of both D-RaCe and SAF in removing filling material from curved root canals previously instrumented (SAF or Revo-S) and filled [cold lateral compaction (CLC) or thermoplasticized injectable techniques (TT)]. The null hypothesis was that there was no difference between the retreatment systems in terms of the canal volume that could be instrumented and filled via different techniques.

## 2. Methods and Materials

### 2.1. Sample Selection

Twenty freshly extracted human mandibular first molars with curved mesial roots were used in this study. Teeth with caries, internal or external resorption, cracks, or immature apices were excluded. The teeth were cleaned of debris and soft tissue remnants and stored in a 0.1% thymol solution until the experiment.

The mesial roots of teeth have two separate canals and apical foramens; thus, 40 root canals with similar curvature were selected. The distal roots were separated and access cavities were prepared with a high-speed bur. A composite filling platform was used as a jig to prepare both the mesial and buccal faces of the tooth to standardize the radiographs. Digital radiographs (Digora; Soredex, Helsinki, Finland) were taken in the buccolingual and mesiodistal directions. Canal curvatures were measured in both directions by two calibrated operators using the methods described by Schneider [18], and mean values for each root canal were calculated. The mean curvature was 26.5° ± 0.3 (*P* = 0.558).

Root canal length was determined with a size 15 K-file (Dentsply Maillefer, Ballaigues, Switzerland) introduced passively into the canal until its tip was just visible at the major apical foramen. Then, using a dental operating microscope, the real canal length was recorded (average length 22 mm) and the working length (WL) was calculated by subtracting 1 mm from this measurement. Forty mesial root canals (20 mesiobuccal and 20 mesiolingual) were instrumented with SAF or Revo-S files, as described later. In the interest of standardization, the mesiobuccal canal was instrumented with SAF and the mesiolingual canal was instrumented with Revo-S in each tooth.

### 2.2. Root Canal Instrumentation

#### 2.2.1. SAF Instrumentation

Twenty root canals were instrumented with the SAF. A glide path was placed using K-files to facilitate the insertion of a size 20 K-file to the WL. SAF files were then operated using an in and out manual motion for 4 min at 5000 vibrations per min. Continuous irrigation with 2.5% NaOCl was provided by a VATEA peristaltic pump (ReDent-Nova) at a rate of 4 mL/min. The final irrigation was performed using a syringe and a needle with 2 mL/min of 17% EDTA, following 2 mL of 2.5% NaOCl.

#### 2.2.2. Revo-S Instrumentation

Twenty root canals were instrumented with Revo-S files. The files were set into permanent rotation with a 6 : 1 contra-angle handpiece (Sirona, Bensheim, Germany) powered by a VDW Gold motor (VDW, Munich, Germany). They were used in a gentle in and out motion with a rotational speed of 300 rpm, according to the manufacturer's instructions. The torque was adjusted to 2.5 Ncm and a crown-down approach was selected. Shaping and cleaning file of SC1 was used to enlarge the coronal two-thirds of the root canal. SC2 and SU files were used at WL. Between each file, 2 mL of 2.5% NaOCl was used, and the final irrigation was performed with 2 mL/min of 17% EDTA following 2 mL of 2.5% NaOCl.

### 2.3. Root Canal Filling

The operator was blinded to the tooth group being filled. The root canals were dried with paper points and filled with gutta-percha and AH Plus (Dentsply De Trey GmbH, Konstanz, Germany) root canal sealer. Prior to use, the sealer was mixed until it reached a thick consistency, in accordance with the manufacturer's instructions.

#### 2.3.1. SAF System + Cold Lateral Compaction Group (SAF + CLC Group)

Ten root canals instrumented with SAF were filled using the CLC technique with a size 30 master cone and accessory gutta-percha cones.

#### 2.3.2. SAF System + Thermoplasticized Injectable Technique Group (SAF + TT Group)

Ten root canals instrumented with SAF were filled using the TT technique. Two mm of a size 30 master cone was placed into the apical part via a finger plugger (VDW) to prevent overfilling of the apex. A cordless backfill filling system (Dia-Gun; DiaDent, Burnaby, BC, Canada) was prepared according to the manufacturer's instructions. The working temperature was adjusted to 180°C. A 23-gauge silver needle was placed within 5 mm of the prepared apical stop and the gutta-percha was expressed. When back pressure was felt, the needle was withdrawn and a finger plugger was used to apply firm apical pressure to the gutta-percha. The remaining root canal space was backfilled until gutta-percha was observed in the orifice and compacted with a hand plugger (Roeko, Langenau, Germany) to finish the filling.

#### 2.3.3. Revo-S + Cold Lateral Compaction Group (Revo-S + CLC Group)

Ten root canals instrumented with Revo-S were filled as in the SAF + CLC Group.

#### 2.3.4. Revo-S + Thermoplasticized Injectable Technique Group (Revo-S + TT Group)

Ten root canals instrumented with Revo-S were filled as in the SAF + TT Group.

Access cavities were filled with a temporary filling material (Fermin; Detax Dental, Karlsruhe, Germany) and then stored for seven days at 37°C and 100% humidity to allow the sealer to set fully.

### 2.4. Retreatment Procedures

Initial reinstrumentation of all root canals was performed in the same manner, as follows: a 3 mm coronal portion of each canal filling was removed with Gates Glidden drills (size 3 and size 2) at 2000 rpm. After using the drills, 0.1 mL of chloroform was introduced into the root canal to soften the gutta-percha for 2 min before further instrumentation. To eliminate interoperator variability, the same operator (NS) carried out all of the intracanal procedures.

All of the root canals were reinstrumented with D-RaCe in the first step phase and then scanned with a micro-CT (Model 1172; Skyscan, Kontich, Belgium).

### 2.5. Micro-CT Scanning Analysis

All teeth were scanned at three stages: after root canal filling, after reinstrumentation with D-RaCe (Step 1), and after reinstrumentation with SAF (Step 2). Each tooth was dried slightly and mounted on a custom attachment, and the analysis of the filling materials was carried out using a micro-CT system. Each specimen was scanned for a total of 60 min. Lengths of the teeth were scanned at 100 kV, 100 *μ*A, and an isotropic pixel size of 13.7 *μ*m, which resulted in 900–1200 transverse cross-sections perspecimen. Scanning was performed with 180° rotations around the vertical axis, camera exposure time of 2800 ms, rotation steps of 0.6°, frame averaging of 2, and medium filtering of the data. X-rays were filtered with 500 *μ*m aluminum and a 38 *μ*m thick copper filter. A flat-field correction was taken on the day prior to scanning to correct for variations in the pixel sensitivity of the camera. Axial cross-sections of the inner structures of the samples were reconstructed using NRecon v.1.6.3 (Bruker microCT) with a beam hardening correction of 35%, smoothing of 4, and an attenuation coefficient range of 0–0.152.

To calculate the volumes of the fillings, the original gray scale images were processed with a slight Gaussian low-pass filtration for noise reduction, and an automatic segmentation threshold was used to separate root dentine from filling and voids, using CTAn v.1.12 software (Bruker-microCT). This process entails choosing the range of gray levels for each filling, dentine, or void, necessary to obtain an image composed only of black and white pixels. The high contrast of the filling compared with the dentine yielded excellent segmentation of the specimens. Separately and for each slice, regions of interest were chosen to allow calculation of the volume (in mm^3^) of the filling and the voids. Polygonal surface representations of dentine, filling, and voids were constructed in CTAn and qualitatively evaluated with CTVol v.2.2.1 software (Bruker-microCT). In this study, all areas without filling within the root canal space after the retreatment procedures were considered voids. Lateral or accessory canals were not considered in the analysis.

**Step 1 (D-RaCe reinstrumentation).**

All of the root canals were reinstrumented with D-RaCe files. The files were set into permanent rotation with a 6 : 1 contra-angle handpiece (Sirona, Bensheim, Germany) powered by a VDW Gold motor. A D-RaCe file (size DR1) at 800 rpm was used in the coronal third of the canal with a crown-down technique. The DR2 file was used with light apical pressure until the WL was reached. The canals were irrigated with 5 mL of 2.5% NaOCl between each instrument. A 5 mL/min final rinse with 17% EDTA was performed following 2 mL of 2.5% NaOCl.

After these procedures, the same root canals were instrumented again with the SAF system in the second step phase and then rescanned.

**Step 2 (SAF reinstrumentation).**

After completion of reinstrumentation with the D-RaCe files, an additional SAF file was positioned into the canal to the WL using an RDT3 handpiece head (ReDent-Nova) at 5000 rpm and amplitude of 0.4 mm. A pecking motion was used, according to the manufacturer's instructions, for 2 min. The SAF was connected to a VATEA system irrigator (ReDent-Nova) at a continuous flow of 2.5% NaOCl at a flow rate of 5 mL/min. A 5 mL/min final rinse with 17% EDTA was performed following 2 mL of 2.5% NaOCl.

### 2.6. Statistical Analysis

The statistical analysis was performed using SPSS software version 13.0 (SPSS Inc., Chicago, IL). All data were reported as mean ± standard deviation. Normality for continued variables in groups was determined by the Shapiro-Wilk test. The variables did not show normal distribution (*P* < 0.05), so Kruskal-Wallis and Wilcoxon tests were used for comparisons among the tested groups. A value of *P* < 0.05 was considered significant.

## 3. Results

**Figure 1:**
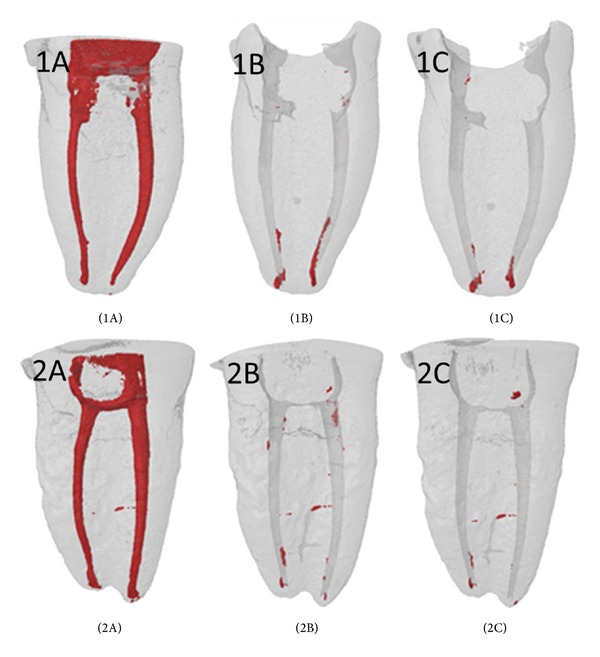
Representative cases of root filling removal. Canals instrumented with SAF (left canal) and Revo-S (right canal). After canal fillings with CLC (1A) and TT (2A) techniques. After reinstrumentation with D-RaCe (Step 1) (1B and 2B). After reinstrumentation with SAF (Step 2) (1C and 2C).

The percentages of filling material removed from the root canals after retreatment are shown in Table 1 and Figure 1. None of the procedures removed all of the remains of the root canal filling material from any of the teeth. There were no statistically significant differences between the filling techniques in the canals instrumented with SAF (*P* = 0.292). Similar results were found in the canals instrumented with Revo-S (*P* = 0.306). According to the Kruskal-Wallis test, the quantity of remaining filling materials was similar in all of the groups (*P* = 0.363). However, the additional use of SAF was more effective than using D-RaCe files alone in achieving cleanliness of the root canals (Table 1 and Figure 1).

## 4. Discussion

Posttreatment disease is likely due to the persistence or emergence of microorganisms in the root canal system after cleaning and shaping or the recolonization of the root canal space by bacteria following microleakage [19, 20]. Removing the etiological factors (necrotic tissues, bacterial biofilms, coronal leakage, recurrent caries, and tooth fractures) results in conditions conducive to healing; thus, nonsurgical endodontic treatment is preferred to periapical surgery for treating persistent infections [7]. The basic goal of nonsurgical endodontic treatment is to reduce or eliminate, to the extent possible, the microbial flora [21]. Removing all root fillings is a prerequisite of nonsurgical retreatment in order to uncover the remnants of necrotic tissue or bacteria that might have caused the previous treatment to fail.

Several destructive and two-dimensional techniques [6, 10, 14, 15] have been used to evaluate the quantity of remaining filling materials after retreatment; however, these methods are not able to evaluate precisely the volume of remaining filling material after the retreatment procedures [22]. Shortcomings of these methods are loss of remaining filling during splitting, variation among different observers due to subjective evaluation, and underestimation of remnants due to two-dimensional imaging [11]. Recently, the micro-CT imaging method (a nondestructive and noninvasive method) has been used to evaluate the efficacy of different retreatment techniques. It allows for the reconstruction and volumetric evaluation of tooth tissues as well as filling materials, overcoming the limitations of conventional methods [22]. For these reasons, the micro-CT imaging method was chosen in this study.

Several studies [23, 24] have explicitly put forth the complexity of the root canal anatomy. Variations in canal sections, in-canal irregularities, and associated curvature diversities render procedure failures almost inevitable. Shaping the canal by preserving its curvature is one of the main parameters used to analyze the methods or instruments developed for root canal preparation. Lee et al. [25] described the features of the mandibular molar mesial roots as curved canals and concave distal surfaces, characteristics that present a potential risk of strip perforations and root fractures. Siqueira et al. [26] stated that the mesial roots of mandibular molars present a high degree of complicity, making it difficult to achieve optimal results in terms of antibacterial and shaping ability. In clinical settings, clinicians often encounter curved root canals, but due to their complex anatomy, not many clinical studies in the literature have included them. In this study, mesial roots of mandibular molars with curved canals were used, under complex anatomic conditions, to test files and filling techniques used in the removal of obturation.

Gutta-percha in conjunction with sealers is the most common root filling because it is inert, usefully plastic when heated, and stable and is tolerated by the tissues. One of the basic properties of an ideal filling is that it should be removable whenever necessary for retreatment purposes [27]. However, inadequately shaped root canals require a substantial effort when performing retreatment [28]. Conventional filling techniques rely on cold or warm compaction of gutta-percha into the canal system. The CLC technique is regarded as a reference when condensing other techniques. However, it has been reported that the quality of adaptation between the canal wall and gutta-percha is uncertain in fillings using the CLC technique [29]. According to several studies [30, 31], warm gutta-percha techniques have exhibited better penetration and sealing ability, while a recent meta-analysis [32] revealed no difference in long-term outcomes when comparing warm condensation and CLC techniques. In the current study, in each mandibular mesial root, the mesiobuccal canals were instrumented with SAF and the mesiolingual canals were instrumented with Revo-S. The canals were then filled with gutta-percha and AH Plus using either the CLC or the TT technique. The results revealed a significantly lower percentage of remaining filling material in samples from the CLC and TT groups. No statistically significant differences were found between filling techniques in the canals instrumented with SAF and Revo-S files. In a recent retreatment study [22], two filling techniques were compared in oval canals, and less filling material was found in the warm vertical condensation (incremental downpack and incremental backfill) group than in the CLC group. The researchers connected this result to higher bond strength, direct contact of the sealer with the dentine, and sealer penetration ability. Unlike that study, we used a modified warm compaction (continuous downpack and continuous backfill) technique, which might explain the difference in the results of the two studies.

The SAF system has a completely different file design and principle of action, and it allows for simultaneous continuous irrigation during instrumentation to facilitate debris and bacteria removal. Recent studies [33, 34] have shown that it is able to touch all of the canal walls evenly. Peng et al. [32] found that operating the SAF with continuous irrigation, using alternating irrigants, resulted in a clean and mostly smear-free dentinal surface in all parts of the root canal. De-Deus et al. [35] compared the debridement efficacy of SAF histologically with rotary instrumentation and demonstrated that it was more efficient in pulpal debridement. Melo Ribeiro et al. [34] reported that SAF removed more debris than rotary instrumentation in the apical third of mandibular incisors. Furthermore, a recent study [14] stated that SAF after using rotary retreatment instrumentation resulted in a significant reduction in the amount of filling residue in curved canals. On the other hand, Paranjpe et al. [36] reported uncontrolled apical instrumentation and inadequate apical irrigation when using the SAF. In actuality, it cannot be considered as a tool to remove fillings [14] or calcium hydroxide [37] on its own; it is not a penetrating instrument and is too flexible to accomplish such a task. Nevertheless, it consists of a metal mesh that is claimed to intimately adapt to the canal walls and have a scrubbing effect [15]. In current retreatment studies [14–16, 22], different two-step protocols with SAF systems were used, according to the wishes of the researchers. The system was used as a supplementary instrumentation system. All of those studies found that additional use of the SAF after using rotary instruments (ProTaper + SAF [14, 15]; ProFile + SAF [17]; R-Endo + SAF [14]) resulted in a significant reduction in the amount of filling residue. In the current study, the additional use of SAF resulted in a significant reduction in the amount of root canal filling residue left after using D-RaCe files.

According to the manufacturer, root canal retreatment with D-RaCe produces efficient removal of the previous filling material. Rödig et al. [11] showed that D-RaCe was significantly more effective than ProTaper retreatment files and hand files in removing filling material from curved canals, and a laboratory study reported that ProTaper retreatment files caused more procedural errors [38]. In contrast, da Silva et al. [4] found D-RaCe to be less effective when used without additional instrumentation. According to the authors, this result might be related to the DR2 file, as it is thinner and has an inactive tip, making it difficult to penetrate the gutta-percha. In the present study, the finding that root canal filling remnants were left in the canal after the first step, in which D-RaCe was used alone, is not surprising. However, by using advanced instrumentation techniques such as SAF after using D-RaCe, curved root canals can be effectively cleaned of the remnants.

## 5. Conclusion

According to the results of this study, neither instrumentation technique nor filling technique affected the retreatment techniques or was significantly different in removing residual filling material from curved root canals. All of the instrumentation techniques left filling residue inside the root canals, but the additional use of SAF was more effective than the use of D-RaCe alone.

## Figures and Tables

**Table 1:** Percentages of removed filling material from root canals after retreatment (mean ± standard deviation).

Groups	*n *	D-RaCe (Step 1) (%)	SAF (Step 2) (%)	*P*
SAF + CLC	10	95.22 ± 3.68	96.6 ± 3.28	0.018
SAF + TT	10	97.99 ± 3.49	98.55 ± 2.94	0.043
Revo-S + CLC	10	97.78 ± 1.43	99.04 ± 0.65	0.008
Revo-S + TT	10	96.67 ± 5.57	97.89 ± 3.93	0.018
